# Investigating the shared genetic architecture between primary sclerosing cholangitis and inflammatory bowel diseases: a Mendelian randomization study

**DOI:** 10.1186/s12876-024-03162-6

**Published:** 2024-02-19

**Authors:** Xuan Dong, Li-Li Gong, Mei-Zhu Hong, Jin-Shui Pan

**Affiliations:** 1 Department of Hepatology, the First Affiliated Hospital of Fujian Medical University, Fuzhou, Fujian China; 2 Hepatology Research Institute, Fujian Medical University, Fuzhou, Fujian China; 3 Department of Hepatology, National Regional Medical Center, Binhai Campus of the First Affiliated Hosptial, Fujian Medical University, Fuzhou, Fujian China; 4 Fujian Clinical Research Center for Hepatopathy and Intestinal Diseases, Fuzhou, Fujian China; 5 Department of General Practice, Zhongshan Hospital, Xiamen University, Xiamen, Fujian China; 6 Department of Traditional Chinese Medicine, Mengchao Hepatobiliary Hospital of Fujian Medical University, Fuzhou, Fujian China

**Keywords:** Mendelian randomization, Primary sclerosing cholangitis, Ulcerative colitis, Crohn’s disease

## Abstract

**Background:**

Several studies have found that primary sclerosing cholangitis (PSC) and inflammatory bowel disease (IBD) are closely associated. However, the direction and causality of their interactions remain unclear. Thus, this study employs Mendelian Randomization to explore whether there are causal associations of genetically predicted PSC with IBD.

**Methods:**

Genetic variants associated with the genome-wide association study (GWAS) of PSC were used as instrumental variables. The statistics for IBD, including ulcerative colitis (UC), and Crohn’s disease (CD) were derived from GWAS. Then, five methods were used to estimate the effects of genetically predicted PSC on IBD, including MR Egger, Weighted median (WM), Inverse variance weighted (IVW), Simple mode, and Weighted mode. Last, we also evaluated the pleiotropic effects, heterogeneity, and a leave-one-out sensitivity analysis that drives causal associations to confirm the validity of the analysis.

**Results:**

Genetically predicted PSC was significantly associated with an increased risk of UC, according to the study (odds ratio [OR] IVW= 1.0014, *P*<0.05). However, none of the MR methods found significant causal evidence of genetically predicted PSC in CD (All *P*>0.05). The sensitivity analysis results showed that the causal effect estimations of genetically predicted PSC on IBD were robust, and there was no horizontal pleiotropy or statistical heterogeneity.

**Conclusions:**

Our study corroborated a causal association between genetically predicted PSC and UC but did not between genetically predicted PSC and CD. Then, we identification of shared SNPs for PSC and UC, including rs3184504, rs9858213, rs725613, rs10909839, and rs4147359. More animal experiments and clinical observational studies are required to further clarify the underlying mechanisms of PSC and IBD.

**Supplementary Information:**

The online version contains supplementary material available at 10.1186/s12876-024-03162-6.

## Introduction

Inflammatory bowel disease (IBD) is a chronic intestinal disorder with unknown etiology. Many studies point to the presence of genetic predisposition, intestinal mucosal immune system dysfunction, and microbiota imbalance [1] in the occurrence and progression of IBD. Ulcerative colitis (UC) and Crohn’s disease (CD) are two main typical subtypes of IBD. The incidence of IBD has risen over the past decade in Asia. Predictably, the prevalence of IBD will significantly in the future, following an aging population [2].

Patients with IBD not only suffer a significant reduction in their quality of life but also causes substantial costs in health care due to its high prevalence [3]. Chronic IBD is restricted to the gut, but also in the extraintestinal organs in many patients [4, 5]. This phenomenon is called extraintestinal manifestations (EIM) of IBD. EIM frequently affects joints [6], skin [7], eyes [8], lungs [9], pancreas [10], and liver [11]. Primary sclerosing cholangitis (PSC) is important EIM in IBD patients [4]. In clinical, about 70% of PSC patients are found to have underlying IBD [12–14]. Genetic risk factors, environmental factors, activation of the immune system, and microbiota have been assumed that the factors relevant to the pathogenesis of EIMs [15, 16]. For PSC, the association with the activity of the underlying IBD is unclear [17].

PSC is a type of autoimmune liver disease characterized by multi-focal bile duct strictures and progressive liver disease [18]. The prognosis of PSC was not satisfactory. Most patients ultimately require liver transplantation, after which disease recurrence may occur. However, without liver transplantation, the median survival time for PSC patients is 10 to 12 years [19].

Similar to IBD, the pathogenesis of PSC is also not well clear. However, the characteristic that PSC is often accompanied by IBD suggests that there may be a shared pathogenic gene or pathway between the PSC and IBD. Mendelian randomization (MR) renders us a novel way to study the connection between these two diseases. MR is a genetic epidemiological method, this method follows the Mendelian genetic law of "parental alleles are randomly assigned to offspring" [20]. This method uses single-nucleotide polymorphisms (SNPs) as instrumental variables (IVs) to infer the potential causality of exposure and outcome. It’s beneficial to minimize bias caused by confounding factors and reverse causality [21]. Based on this, the MR method has been widely used to assess the causal relationship between traits and diseases or between diseases [22–24]. To the best of our knowledge, there are no MR studies inferring the potential causal relationship of PSC with IBD to date. Therefore, we applied the MR method to examine whether the genetically predicted PSC is associated with IBD.

## Methods

### Study design

**Fig. 1:**
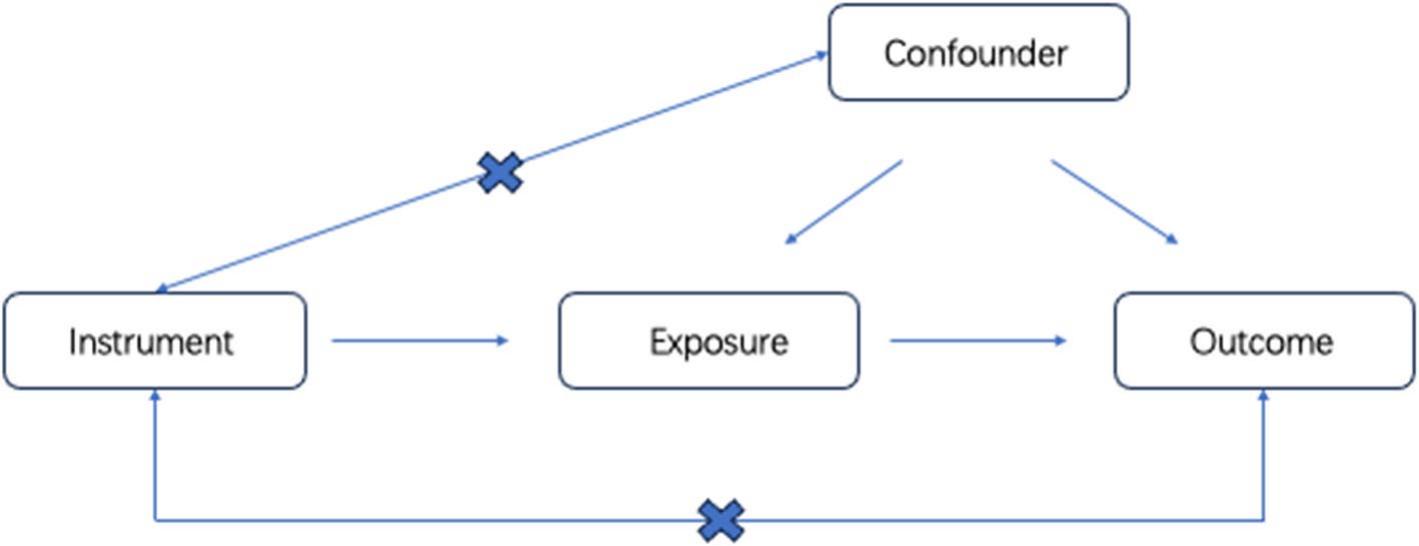
Study design and workflow in the present study

The overall design of Mendelian randomization analyses in this study is shown in Fig. 1. Briefly, (1) The selected instrumental variables were linked to exposure; (2) there were no inherent interactions between instrumental variables and confounder factors; (3) exposure was the only way by which instrumental variables can affect outcomes. The PSC served as the exposure, and UC served as the outcome. Since all datasets used in this study were based on public databases, no additional ethical approval was required.

### GWAS data for PSC, UC, and CD

We gathered the summary statistics of PSC, UC, and CD, from the IEU Open GWAS project (https://gwas.mrcieu.ac.uk/), all the cases there were defined on the basis of the International Classification of Diseases (ICD), and fulfilled the clinical diagnosis criteria for IBD and PSC. To be more specific, the sample sizes of datasets for PSC, UC, and CD, are 14,890 cases, 463,010 cases, and 212,356 cases, respectively. PSC has 7, 891, 603 SNPs, UC has 9, 851, 867 SNPs, and CD has 16, 380, 455 SNPs. The detailed information on GWAS data is shown in Table 1.

**Table 1:** Detailed information on association studies in our analysis

	Year	ID	Population	Sample size	
				Cases	SNPs
UC	2018	ukb-b-7421	European	463,010	9,851,867
CD	2021	finn-b-K11_CROHN	European	212,356	16,380,455
PSC	2017	ieu-a-1112	Mixed	14,890	7,891,603

*UC* Ulcerative colitis, *CD* Crohn’s disease, *PSC* Primary sclerosing cholangitis, *SNPs* Single nucleotide polymorphisms

### Instrumental variable selection

All statistical analyses were performed by the R packages: TwoSampleMR. First, we selected SNPs related to PSC at the genome-wide significance threshold with *p*< 5 × 10^-8^. Because strong linkage disequilibrium could lead to biased results. Second, the independence among the selected SNPs was evaluated according to the pairwise-linkage disequilibrium (r2 < 0.001, clumping window of 10,000 kb). When F-statistics were greater than 10, SNPs were considered powerful enough to mitigate the influence of potential bias. Third, we selected SNPs with F statistic >10 as IVs.

### Statistical analysis

Based on the IVs, we performed an MR analysis to investigate the relationship between PSC and IBD. Five popular MR methods were used to analyze our data: MR Egger, Weighted median (WM), Inverse variance weighted (IVW), Simple mode, and Weighted mode. The IVW method is reported to be slightly more powerful than the others under certain conditions.

Cochran's Q statistics were used to perform heterogeneity, and *p* > 0.05 indicated no heterogeneity. Moreover, the MR-Egger method was used to determine the horizontal pleiotropy, MR-Egger at a *p*-value < 0.05 can imply the presence of horizontal pleiotropy.

## Results

### Selection of instrumental variables

After a series of quality control steps as mentioned above, 18 SNPs were selected as IVs (Table 2).

**Table 2:** Detailed information for the SNPs in MR analysis

SNPs	CHR	Position	Nearest gene	EA	*P* value
rs10909839	1	2708430	TTC34	A	3.16E-08
rs72837826	2	111933001	-	T	1.26E-09
rs231389	2	204634730	-	T	4.42E-09
rs80060485	3	71153890	FOXP1	C	8.54E-09
rs9858213	3	49731861	RNF123	T	2.43E-20
rs13119723	4	123218313	KIAA1109	G	2.22E-10
rs139010734	6	31974014	CYP21A1P	T	1.98E-154
rs34645399	6	32589169	-	G	1.63E-59
rs3131781	6	30937732	-	G	1E-200
rs114581973	6	33064950	-	T	3.4E-08
rs41316239	6	32779280	-	A	4.97E-11
rs4147359	10	6108439	-	A	4.06E-13
rs79940565	11	63560994	-	C	2E-08
rs3184504	12	111884608	SH2B3	C	5.05E-10
rs725613	16	11169683	CLEC16A	G	5.5E-10
rs313839	19	47221557	PRKD2	G	2.12E-08
rs4817988	21	40468838	-	A	4.2E-15
rs145832854	22	25310129	SGSM1	A	2.58E-08

*SNPs* Single nucleotide polymorphisms, *CHR* Chromosome, *EA* EFFECT allele

### Causality relationship between PSC and IBD

Among the five MR methods, the causal effects of genetically predicted PSC on UC and CD were inconsistent. The results of the MR analyses were shown in Table 3, genetically predicted PSC was positively associated with a risk of UC in our study, with a *p*-value of IVW method less than 0.05. However, we found no evidence supporting a causal association between PSC and CD. Previous research indicated that the genome-wide genetic correlation between PSC and UC was significantly greater than that between PSC and CD [25], similar to our results.

**Table 3:** Association of genetically predicted PSC with risk of UC and CD

Exposure	Outcome	Method	SNPs	b	se	*P* value	OR (95% CI)
Primary sclerosing cholangitis	Ulcerative colitis	MR Egger	5	0.002156613	0.001954797	0.350475456	1.0022 (0.9983–1.0060)
		Weighted median	5	0.00157667	0.00033714	2.91671E-06	1.0016 (1.0009–1.0022)
		Inverse variance weighted	5	0.001395479	0.000285331	1.00457E-06	1.0014 (1.0008–1.0020)
		Simple mode	5	0.001674616	0.000507346	0.029912705	1.0017 (1.0007–1.0027)
		Weighted mode	5	0.001723726	0.000477383	0.022540952	1.0017 (1.0008–1.0027)
Primary sclerosing cholangitis	Crohn’s disease	MR Egger	17	-0.097709	0.06603742	0.15967422	0.9069 (0.7968–1.0322)
		Weighted median	17	-0.0306259	0.04773732	0.52116485	0.9698 (0.8832–1.0650)
		Inverse variance weighted	17	0.08913064	0.04867668	0.0670894	1.0932 (0.9937–1.2027)
		Simple mode	17	-0.002681	0.12079877	0.98256754	0.9973 (0.7871–1.2638)
		Weighted mode	17	-0.0388213	0.04548044	0.40593454	0.9619 (0.8799–1.0516)

*SNPs* Single nucleotide polymorphisms, *OR* Odds ratio, *CI* Confidence interval

**Fig. 2:**
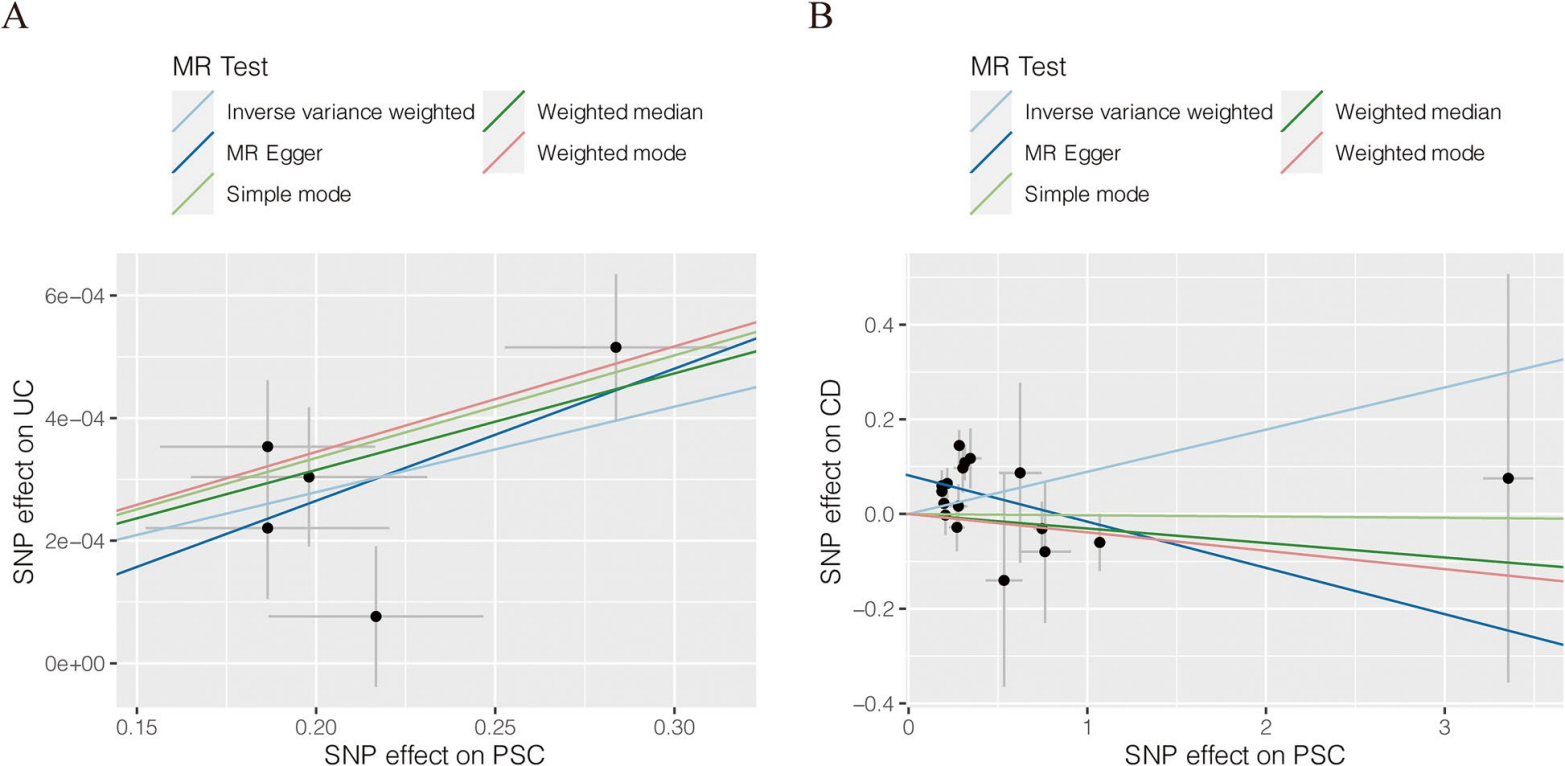
Scatter plots for MR analyses of the causal effect of PSC on UC and CD. **A** PSC against UC. **B** PSC against CD

The scatter plots were used to show the single SNP effect and the combined effects of each MR method (Fig. 2). Forest plots and funnel plots of the causal effect are shown in Supplementary Figure 1.

According to this study, rs3184504, rs9858213, ﻿rs725613, ﻿rs10909839, and ﻿rs4147359 are shared SNPs for PSC and UC (Table 4).

**Table 4:** The shared SNPs for PSC and UC

Nearest genes	SNPs	CHR	BP	EA	OA
SH2B3	rs3184504	12	111884608	C	T
RNF123	rs9858213	3	49731861	T	G
CLEC16A	rs725613	16	11169683	G	T
TTC34	rs10909839	1	2708430	A	G
-	rs4147359	10	6108439	A	G

*SNPs* Single nucleotide polymorphisms, *CHR* Chromosome, *BP* Base pair position, *EA* Effect allele, *OA* Other alleles

### Sensitivity analysis

We performed a leave-one-out sensitivity analysis, heterogeneity, and horizontal pleiotropy to further verify the reliability of our results. The results of sensitivity analysis showed that the causal effect estimation of this study was robust. The MR-Egger (Q *p*-value 0.137) and IVW methods (Q *p*-value 0.214) showed no statistical heterogeneity. Furthermore, no statistical horizontal pleiotropy was found in the horizontal pleiotropy of MR-Egger methods (*P*=0.719). ﻿The results of the sensitivity analysis are shown in Supplementary Figure 2.

## Discussion

The etiology of PSC and IBD remains unclear, and there is a lack of effective treatment methods. Now, the main treatment methods for PSC include bile composition modulators, immune modulators, anti-fibrotic, and regulation of the microbiome. However, further research is needed to determine whether these methods can delay its progression or improve transplant-free survival [26]. The same applies to the treatment of IBD. Although some new methods such as fecal transplantation, and small molecule drugs, applied to the treatment of IBD, satisfactory results have not been achieved in clinical yet [27–29]. Therefore, it is crucial to investigate the relationship between PSC and the subtype of IBD.

Previous studies have suggested an association between IBD and PSC or PBC [30, 31]. PSC is a prototypic gut-liver axis disease. In the patients of PSC, gut microbiota could disrupt the intestinal barrier, leading to bacterial translocation and Th17 cell-driven liver damage [32]. In contrast, the bile acid metabolizing enzyme CYP8B1 inhibits self-renewal of crypt based intestinal stem cells through the accumulation of its product bile acid, hinders intestinal epithelial barrier repair, and exacerbates inflammatory response [33]. These studies indicated a close correlation between intestinal diseases and liver diseases. As mentioned earlier, genetic predisposition plays a role in the occurrence and progression of IBD and PSC. The formation of serum antibodies is a way in which genetic factors affect the immune system. Multiple antibodies such as anti-Saccharomyces cerevisiae antibodies (ASCA), anti-neutrophil cytoplasm antibodies (ANCA) were upregulated in both autoimmune live diseases and IBD [34–36], and those antibodies may predict development of disease. The expression of common antibodies can also indicate a close relationship between the two diseases [37].

In this study, we used GWAS data to investigate the possible causal relationship and specific SNPs between PSC and IBD susceptibility, offering novel insights into the prevention and treatment of PSC and IBD. Multiple MR methods were employed to investigate the relationship between PSC and UC or CD, respectively. Four MR methods (Weighted median, Inverse variance weighted, Simple mode, and Weighted mode) indicated a significant relationship between PSC and UC. However, as for CD, there was no significant relationship between PSC and CD. Thus, we conclude that PSC has a significant relationship with UC but not CD. According this analysis, we also found the specific SNPs that are shared for PSC and UC (rs3184504, rs9858213, ﻿rs725613, ﻿rs10909839, and ﻿rs4147359). Except for chromosome 10 SNP (rs4147359), other SNPs have corresponding genes.

According to a previous study, the chromosome 12 SNP (rs3184504) was in the SH2B3 (SH2B adaptor protein 3) gene and is associated with autoimmune disease [38]. Multiple studies indicated that SH2B3 was related to the occurrence of autoimmune Hepatitis [39–42]. In addition, recent studies have shown that SH2B3 expressed in lymphocytes might with the risk of mid/long-term clinical relapse after being treated with infliximab in those patients with CD [43]. Although how SH2B3 mediates autoimmune disease remains unclear, a study provides us with new insights. Microbiome could exert physiological functions via the SH2B3 gene [44], and gut microbiota also exerts a significant influence on both PSC and UC [45, 46].

And rs9858213 is in the ring finger protein 123 (RNF123) gene, located in chromosome 3. The protein encoded by this gene displays E3 ubiquitin ligase activity toward the cyclin-dependent kinase inhibitor 1B which is also known as p27 or KIP1, so the research on this gene is mainly focused on tumors now [47, 48]. A report indicated that p21 expression was higher in IBD cases [49]. Unfortunately, no studies have been reported that the relationship between rs9858213 and PSC.

T cells play an important role in both PSC and UC. Many studies focus on T-cell immunotherapy [50–53]. C-type lectin domain containing 16A (CLEC16A) gene which, has been proven associate with multiple immune-mediated diseases, which may through T cells to induce pathogenicity [54]. This connection validates our results from an immunological perspective.

﻿For rs10909839, this SNP is located in the tetratricopeptide repeat domain 34 (TTC34) gene. TTC34 gene a link with systemic lupus erythematosus was reported by some studies [55, 56]. Unfortunately, there is limited research on this gene. Therefore, how TTC34 the immune system remains unknown.

We also acknowledge some of the limitations of this study. First, due to data availability, the GWAS data of UC and CD we used were from a European population, while the data of PSC was from a mixed population. In the future, more populations should be included. Second, only 18 SNPs meet the conditions to become IVs. Even if removing linkage disequilibrium, detecting pleiotropy, leave-one-out sensitivity analysis, heterogeneity analysis, and horizontal pleiotropy analysis have been conducted, we cannot guarantee that each SNP site meets the condition that instrumental variables can affect outcomes only through exposure. Some influence of unknown possible confounders inevitably affects our results. We obtained those results by analyzing data from public databases, but the databases didn’t provide clinical data. Therefore, experimental or other studies should be conducted to our results. Despite these limitations, our results may inspire possible mechanism analyses and the relationship between PSC and IBD, in the future.

## Conclusions

Our study corroborated a causal association between genetically predicted PSC and UC but not for PSC and CD. Then, we identification of shared SNPs for PSC and UC, including ﻿rs3184504, rs9858213, ﻿rs725613, ﻿rs10909839, and ﻿rs4147359.

### Supplementary Information

**Supplementary file 1.** 

## Data Availability

All the data used in this study can be downloaded from the IEU Open GWAS project (https://gwas.mrcieu.ac.uk/).
