# Supplementary material for: Investigating the shared genetic architecture between primary sclerosing cholangitis and inflammatory bowel diseases: a Mendelian randomization study
Source: BMC Gastroenterol. 2024 Feb 19;24:77. doi: 10.1186/s12876-024-03162-6 (PMC10875759; doi:10.1186/s12876-024-03162-6)
Supplement: Supplementary file 1 [file 12876_2024_3162_MOESM1_ESM.zip › Supplementary.docx]

## Investigating the shared genetic architecture between multiple Primary sclerosing cholangitis and inflammatory bowel diseases: A Mendelian randomization study

Xuan Dong, Li-Li Gong, Mei-Zhu Hong, Jin-Shui Pan

﻿The first two authors Xuan Dong and Li-Li Gong contributed equally to this paper.

Department of Hepatology, the First Affiliated Hospital of Fujian Medical University, Fuzhou, Fujian, China; Xuan Dong, Jin-Shui Pan

Hepatology Research Institute, Fujian Medical University, Fuzhou, Fujian, China; Xuan Dong, Jin-Shui Pan

Department of Hepatology, National Regional Medical Center, Binhai Campus of the First Affiliated Hosptial, Fujian Medical University, Fuzhou, China; Xuan Dong, Jin-Shui Pan

Fujian Clinical Research Center for Hepatopathy and Intestinal Diseases, Fuzhou, Fujian Province, China; Xuan Dong, Jin-Shui Pan

Department of General Practice, Zhongshan Hospital, Xiamen University, Xiamen, Fujian Province, China; Li-Li Gong

﻿Department of Traditional Chinese Medicine, Mengchao Hepatobiliary Hospital of Fujian Medical University, Fuzhou, Fujian, China; Mei-Zhu Hong

﻿

* Corresponding authors Addresses: Department of Hepatology, the First Affiliated Hospital of Fujian Medical University, Fuzhou, Fujian, China; Hepatology Research Institute, Fujian Medical University, Fuzhou, Fujian, China; No. 20, Chazhong Road, Fuzhou, Fujian 350005, China, Tel.: +86-591-8798-1658. (J.-S. Pan);

or Department of Traditional Chinese Medicine, Mengchao Hepatobiliary Hospital of Fujian Medical University, No. 312, Xihong Road, Fuzhou, Fujian 350025, China;

Tel.: +86-591-8811-6080 (M.-Z. Hong).

﻿E-mail addresses: 546777397@qq.com (M.-Z. Hong), j.s.pan76@gmail.com (J.-S. Pan).

**Supplementary Figure**

**
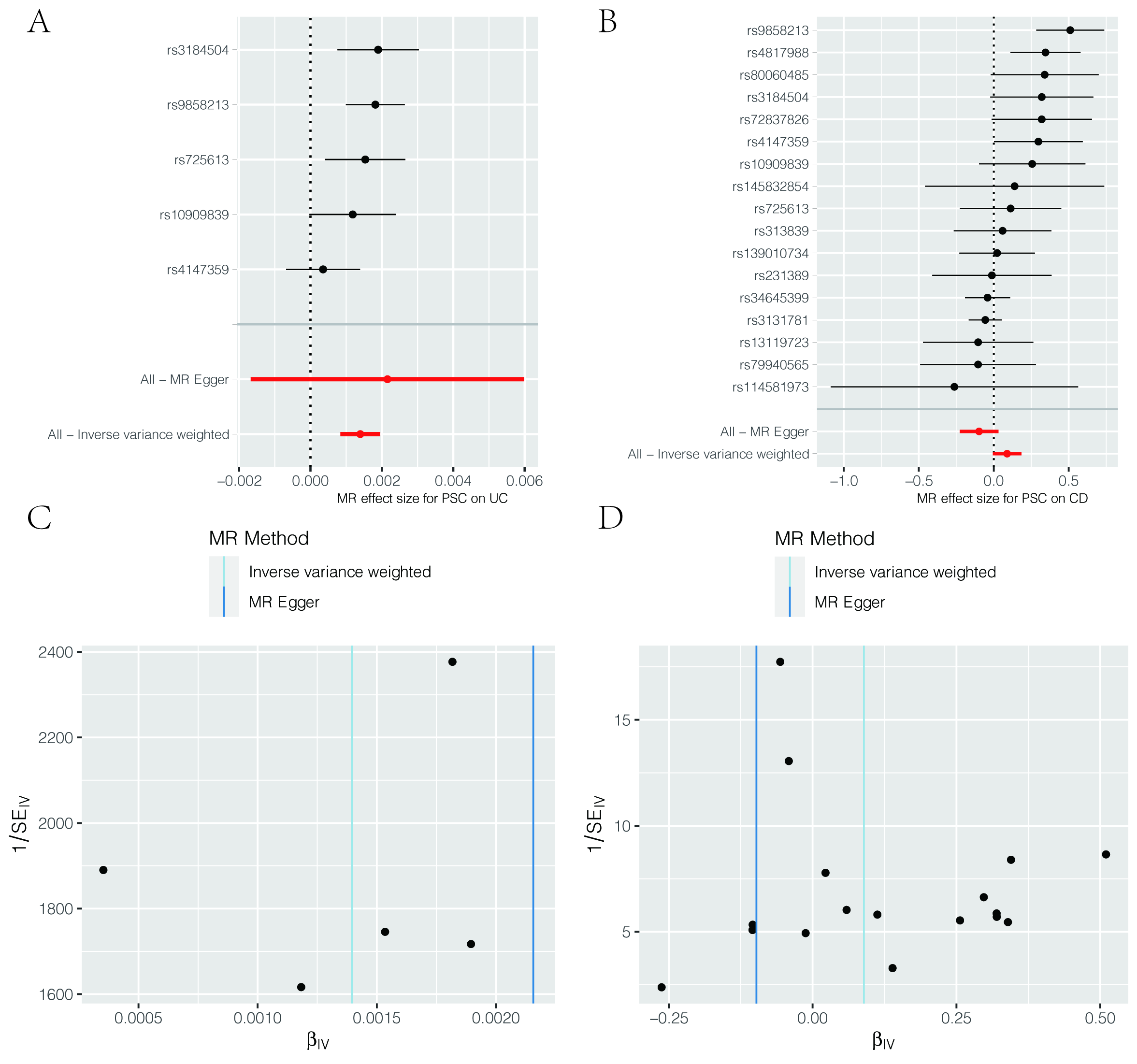
**

Supplementary Figures 1. (A) ﻿Forest plots of the causal effect of genetically predicted PSC on UC. (B) ﻿Forest plots of the causal effect of genetically predicted PSC on CD. (C) Causal relationships between PBC with UC in funnel plots. (D) Causal relationships between PBC with CD in funnel plots.

**
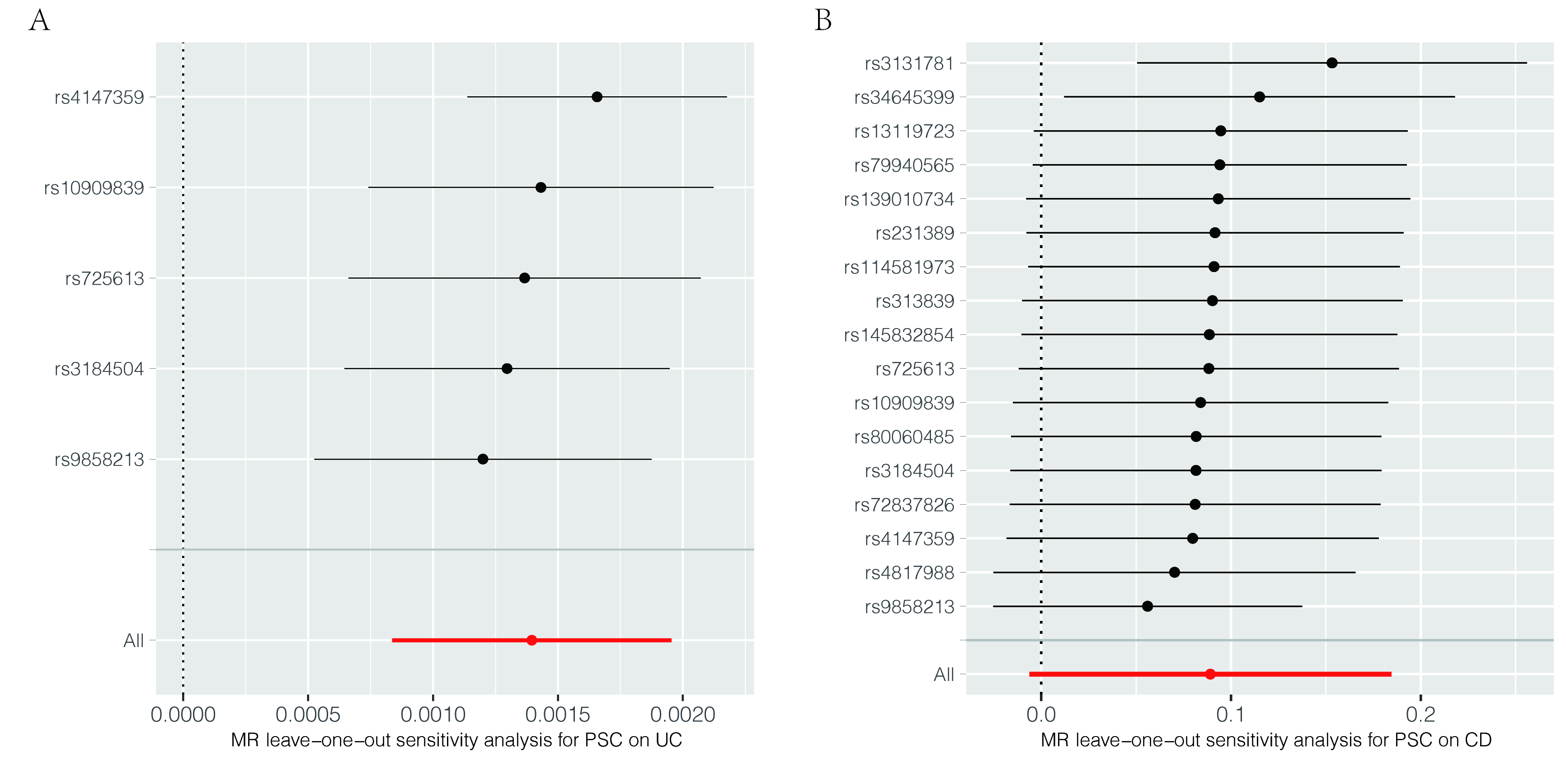
**

Supplementary Figure 2. The results of the leave-one-out sensitivity analysis. ﻿(A) Forest plots of SNPs associated with PSC and UC. ﻿(B) Forest plots of SNPs associated with PSC and CD.
